# Supplementary material for: Novel Starter Strain Enterococcus faecium DMEA09 from Traditional Korean Fermented Meju
Source: Foods. 2023 Aug 9;12(16):3008. doi: 10.3390/foods12163008 (PMC10453556; doi:10.3390/foods12163008)
Supplement: Supplementary file 1 [file foods-12-03008-s001.zip › foods-2512778-supplementary.pdf]

Figure S1. Lipase activity of *Enterococcus faecium* DMEA09 on media supplement NaCl.

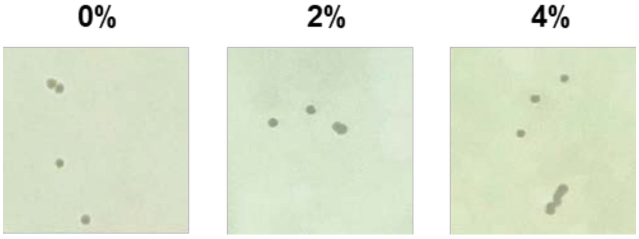

Table S1. Osmotic stress synthesis and transporter system-related genes in the genomes

	Gene	E.C. No.	Product	Gene locus
Inorganic solute				
Transporter	*ybaL*	-	Potassium transporter	PAN98_RS00850
	trkH	-	Ktr system potassium uptake protein D	PAN98_RS03485
	trkH	-	Trk family potassium uptake protein	PAN98_RS10240
	trkA	-	TrkA family potassium uptake protein	PAN98_RS08815
Organic solute				
Transporter	*glpF*	-	Glycerol uptake facilitator and related aquaporins	PAN98_RS01735
	glpF	-	Glycerol uptake facilitator and related aquaporins	PAN98_RS00640
	opuCA	-	ABC transporter permease	PAN98_RS00975
	opuCB	-	ABC transporter permease	PAN98_RS00965
	opuCC	-	Betaine/proline/choline family ABC transporter ATP-binding protein	PAN98_RS00980
	opuCD	-	Osmoprotectant ABC transporter substrate-binding protein	PAN98_RS00970
	opuD	-	ABC transporter permease	PAN98_RS07690
	citS	2.7.13.3	Citrate-sodium symporter	PAN98_RS07595
	gltK		Glutamate/aspartate transport system permease protein	PAN98_RS06395
Synthesis	*gk*	2.7.1.30	Glycerol kinase	PAN98_RS01725
	gpsA	1.1.1.94	NAD(P)H-dependent glycerol-3-phosphate dehydrogenase	PAN98_RS03875
	plsY	2.3.1.15	Glycerol-3-phosphate 1-O-acyltransferase PlsY	PAN98_RS07775
	plsC	2.3.1.51	1-Acyl-sn-glycerol-3-phosphate acyltransferase	PAN98_RS10870
	cdsA	2.7.7.41	phosphatidate cytidylyltransferase	PAN98_RS05230
	pgsA	2.7.8.5	CDP-diacylglycerol--glycerol-3-phosphate 3-phosphatidyltransferase	PAN98_RS11755
	pgpA	3.1.3.27	Phosphatidlyglycerophosphatase	PAN98_RS09790
	clsA	2.7.8.-	Cardiolipin synthase	PAN98_RS03805
	clsA	2.7.8.-	Cardiolipin synthase	PAN98_RS08125
	maeN		Damage-inducible protein CinA	PAN98_RS03315
	proB	2.7.2.11	Glutamate 5-kinase	PAN98_RS11840
	proA	1.2.1.41	Glutamate-5-semialdehyde dehydrogenase	PAN98_RS11845
	proC	1.5.1.2	Pyrroline-5-carboxylate reductase	PAN98_RS11735
